# Forgot to Exercise? Exercise Derived Circulating Myokines in Alzheimer's Disease: A Perspective

**DOI:** 10.3389/fneur.2021.649452

**Published:** 2021-06-30

**Authors:** Rajesh Gupta, Rizwan Khan, Constanza J. Cortes

**Affiliations:** ^1^Department of Cell, Developmental and Integrative Biology (CDIB), School of Medicine, University of Alabama at Birmingham, Birmingham, AL, United States; ^2^Center for Neurodegeneration and Experimental Therapeutics (CNET), University of Alabama at Birmingham, Birmingham, AL, United States; ^3^Center for Exercise Medicine, School of Medicine, University of Alabama at Birmingham, Birmingham, AL, United States; ^4^UAB Nathan Shock Center for the Excellence in the Study of Aging, University of Alabama at Birmingman, Birmingham, AL, United States

**Keywords:** exercise, aging, myokines, exerkines, neuroprotection

## Abstract

Regular exercise plays an essential role in maintaining healthy neurocognitive function and central nervous system (CNS) immuno-metabolism in the aging CNS. Physical activity decreases the risk of developing Alzheimer's Disease (AD), is associated with better AD prognosis, and positively affects cognitive function in AD patients. Skeletal muscle is an important secretory organ, communicating proteotoxic and metabolic stress to distant tissues, including the CNS, through the secretion of bioactive molecules collectively known as myokines. Skeletal muscle undergoes significant physical and metabolic remodeling during exercise, including alterations in myokine expression profiles. This suggests that changes in myokine and myometabolite secretion may underlie the well-documented benefits of exercise in AD. However, to date, very few studies have focused on specific alterations in skeletal muscle-originating secreted factors and their potential neuroprotective effects in AD. In this review, we discuss exercise therapy for AD prevention and intervention, and propose the use of circulating myokines as novel therapeutic tools for modifying AD progression.

## Pathophysiology of Alzheimer's Disease

Alzheimer's disease (AD) is the most prevalent neurodegenerative disease affecting more than 10% of the human population over the age of 65 (1). AD is characterized by impaired executive function, language, and visual processing, eventually leading to dementia, memory loss and circadian and metabolic alterations (1). Pathologically these symptoms are associated with a loss of synapses, neurons, and an overall reduction of gray matter in vulnerable brain regions, including the hippocampus and the cortex. AD is also associated with two classical histopathological hallmarks: first, the accumulation of extracellular neuritic plaques, which are deposits of varied sizes of a small peptide known as amyloid-β (Aβ), synthesized through sequential proteolytic cleavages of the amyloid-β precursor protein (APP). Second, neurofibrillary tangles (NFTs) constituted of hyperphosphorylated microtubule-associated protein tau within affected neurons. These pathological hallmarks accumulate in affected brain areas, including the hippocampus and neocortical regions, and can be detected in the CNS decades before the onset of clinical symptoms (1). Currently there is no cure for AD and most available therapeutics focus on treating symptoms. (1). With estimates predicting around 12 million U.S. cases and 81 million worldwide by 2040 if preventive therapies are not found, the looming public health crisis stresses the urgency to develop novel interventions to prevent or delay the onset and progression of AD.

## Exercise and Neuroprotection

**Figure 1:**
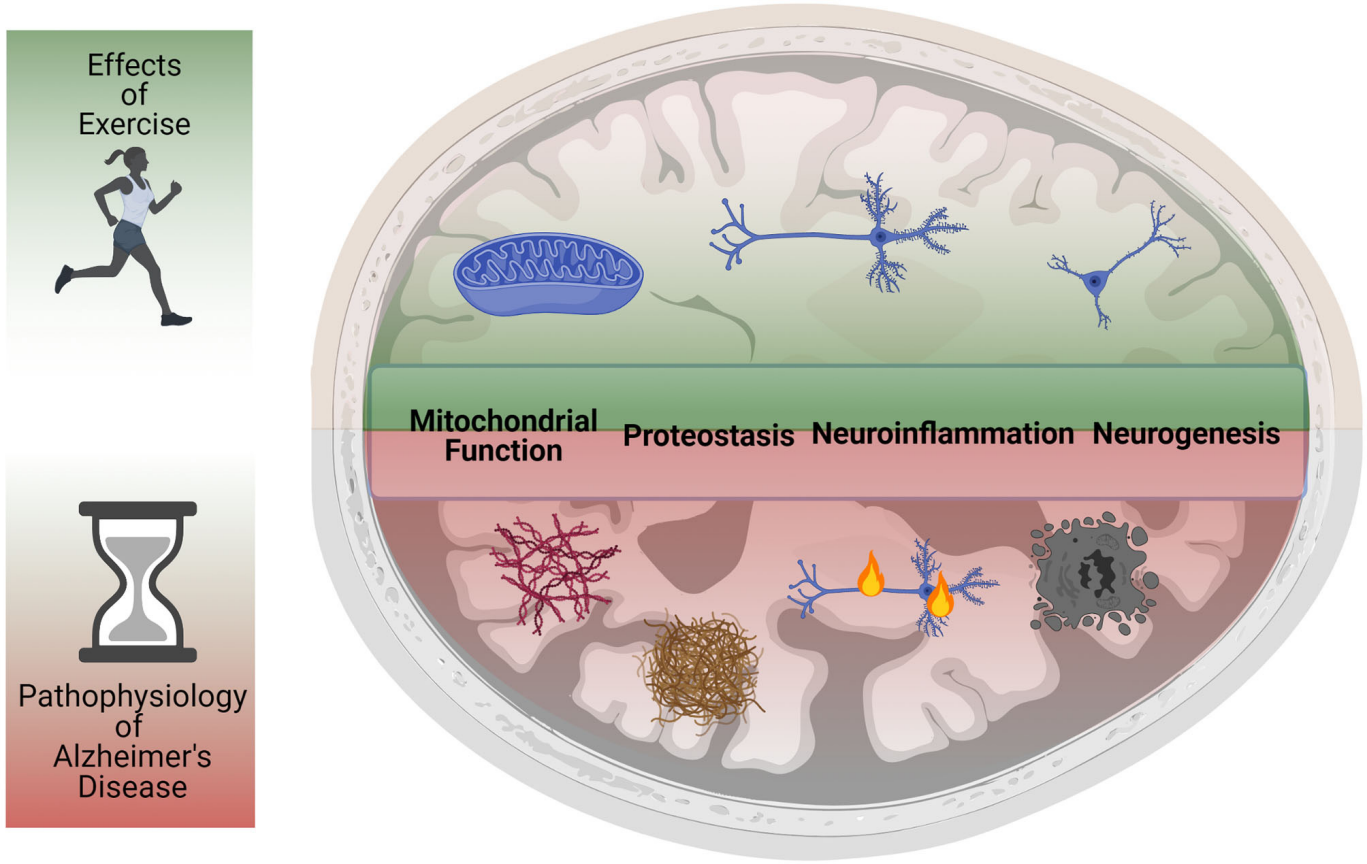
Exercise reduces primary and secondary pathological hallmarks of Alzheimer's Disease. Studies in humans and animal models have shown that physical activity and exercise can have powerful protective effects in the CNS. Exercise reduces neuroinflammation, increases mitochondrial function, improves proteostasis, and rescues impaired neurogenesis. Animal model studies have also demonstrated that exercise reduces the levels of amyloid beta plaques and neurofibrillary tangles, preventing neurodegeneration and preserving cognitive function. The ability of exercise to improve most (if not all) of the pathological hallmarks of AD suggests it may be a powerful intervention against the onset and progression of AD.

Regular exercise plays an essential role in maintaining healthy neurocognitive function and immuno-metabolism in the aging central nervous system (CNS), benefitting cognition and memory during healthy aging (2, 3) and reducing the risk of age-associated neurodegenerative disease (4, 5). Indeed, multiple studies in rodent models have demonstrated that exercise, including forced treadmill and voluntary wheel running, powerfully stimulates adult hippocampal neurogenesis (4, 6–8) and increases brain derived neurotrophic factor (BDNF) levels in the aging hippocampus (7–9). Indeed, the CNS undergoes a progressive functional decline during aging, including increased neuroinflammation and dysfunction of proteostasis and mitochondrial systems (10–12), all of which are believed to contribute to the development of AD and can be modified through exercise interventions (Figure 1). This strongly implicates exercise as a highly relevant behavioral strategy for preventing age-related cognitive decline (3, 4, 8). Although healthy aging and AD are two distinct multifactorial processes (1, 12), aging does remain the highest risk factor for the development of AD (3). This suggests that strategies that bolster CNS resilience during aging may also have important benefits in the prevention of and intervention against AD.

Moreover, physical activity decreases the risk of developing AD (13–15), is associated with better AD prognosis (16), and positively affects cognitive function in AD patients (17, 18). Indeed, individuals that participated in consistent physical activity in midlife reduced their risk for Alzheimer's disease by half (19), highlight the potent neuroprotective effects of exercise. Similar benefits have been reported in AD transgenic mice, where exercise rescues impaired neurogenesis, enhances synaptic plasticity, and attenuates AD associated-neuropathology (Figure 1) (20–25). However, the precise mechanisms responsible for this exercise-dependent rejuvenation of the aging and/or AD CNS remain largely unexplored.

Physical activity can include occupational, sports, conditioning, household, or other activities. Exercise is a subset of physical activity that is planned, structured, and repetitive, with the ultimate objective of improving or maintaining physical fitness (26). Endurance exercise training (running, dancing, cycling), leads to adaptations in both the cardiovascular and musculoskeletal system, supported by muscle hypertrophy and local increases in skeletal muscle mitochondrial biogenesis, capillary density, and oxidative capacity. Resistance exercise (strength training), on the other hand, mostly elicits changes in size and contractile properties of muscle, with direct adaptations on the neuromuscular system which support maximal motor unit synchronization and muscle activation. Since most of the CNS benefits observed in human studies appear to occur after endurance training, suggesting differential biochemical and physiological responses to each exercise intervention (27), we will focus on the effects of this training modality in AD in this review.

## Skeletal Muscle Is an Endocrine Organ Secreting CNS-Targeting “MYOKINES”

**Figure 2:**
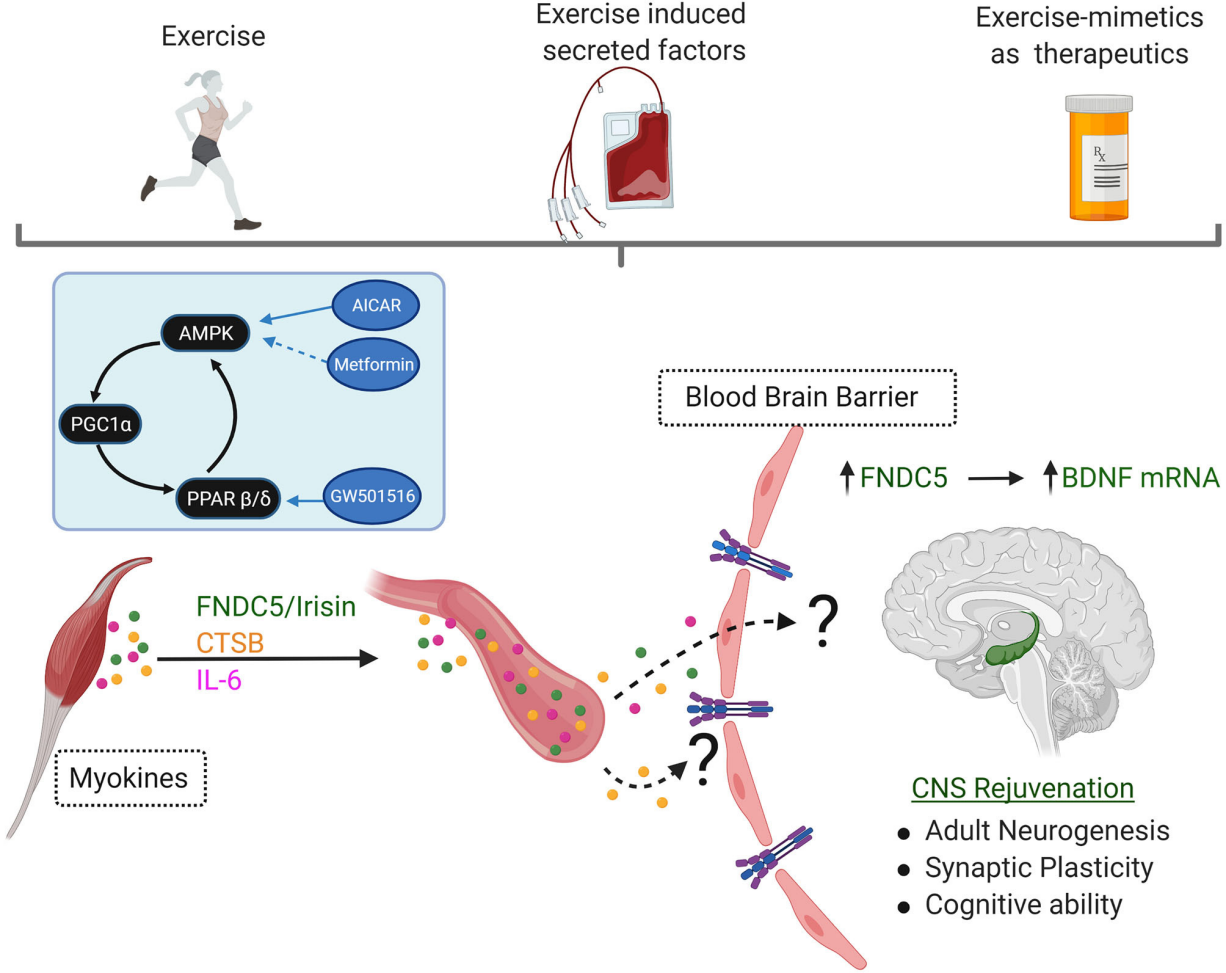
Exercise is a Powerful Behavioral Intervention Against CNS Aging and Alzheimer's Disease. Multiple studies have shown that exercise can have potent rejuvenating effects in the CNS. Increases in secretion of CNS-targeting myokines such as FNDC5/Irisin, Cathepsin B (CSTB), and interleukin-6 (IL-6) are seen in response to exercise. Although the mechanisms (if any) of transport across the blood-brain barrier remain currently poorly understood, they all culminate in increased expression of Brain Derived Neurotrophic Factor (BDNF) in the hippocampus. This elevation in BDNF is required for the neurocognitive benefits of exercise, including increases in adult hippocampal neurogenesis, improvements in synaptic plasticity, increased angiogenesis, and finally improvement or maintenance of cognitive ability. Plasma transfer experiments have demonstrated the existence of these factors in circulation, and their ability to transfer the neurocognitive benefits of exercise in sedentary animal models. Development of therapeutics that reproduce the CNS rejuvenation effects of exercise (exercisemimetics) should perhaps recapitulate not just the local skeletal muscle effects of exercise, but also the exerkine profile seen in circulation. (Inset): Exercise stimulates the AMPK and PGC1α which can work synergistically with PPARβ/δ. This pathway is under intense scrutiny as a potential target for therapeutics that mimic the benefits of behavioral exercise may serve as a neuroprotectant signaling pathway, including AICAR, Metformin, and GW501516.

Skeletal muscle has been proposed as a central regulator of organismal metabolism, communicating metabolic and proteostasis stress to distant tissues including brain via secretion of circulating “myokines” (28–30). In agreement with this, multiple epidemiological studies suggest that skeletal muscle aging is a risk factor for the development of age-associated disease (28, 31), including those of the CNS (5, 32). Skeletal muscle undergoes prominent remodeling during aging (33–35), and exercise powerfully opposes these local deleterious effects, activating metabolic (31, 36) and proteostasis pathways to accommodate increased bioenergetic demands (37). Exercise also increases secretion of specific CNS-targeting myokines (“exercise induced myokines”) into circulation, including BDNF (7), FNDC5/irisin (38), and cathepsin B (39), which very likely contribute to the exercise-associated benefits on cognition (39) (Figure 2). Indeed, shared circulation and young plasma injection experiments have suggested that circulating exercise-induced myokines can promote functional rejuvenation of the aging neurogenic niche (11, 40). Consistent with this hypothesis, circulating blood factors in plasma from exercised aged mice are sufficient to transfer the effects of exercise on adult neurogenesis and cognition to sedentary aged mice (41) (Figure 2). Furthermore, conditioned media from activated muscle cells induces differentiation of adult neural stem cells *in vitro* (42), directly linking muscle originating secreted factors to the function of the aging CNS. Studies in Drosophila have also demonstrated a novel role for skeletal muscle in protecting the brain from age-related proteostasis collapse (43) and the role of nutrient-sensing myokines in regulating dopamine production in the CNS (44). However, to date, our knowledge of circulating myokines and their role in AD remains largely unexplored.

One consistent molecular biomarker of exercise in the brain is the induction of neurotrophins, particularly BDNF. BDNF promotes multiple aspects of CNS biology, including neuronal survival and differentiation, synaptogenesis, and synaptic plasticity. BDNF expression is robustly induced in the hippocampus with exercise (45), and active BDNF signaling is required for the exercise-associated benefits on memory and learning (46). However, the underlying mechanisms inducing BDNF activation in the hippocampus in response to exercise remain largely unknown. Recently, several exercise-associated myokines have arisen as novel regulators of BDNF activity during exercise (Figure 2). In this section, we discuss our current limited understanding of exercise-responsive skeletal muscle secreted factors and their effects on hallmarks of CNS function and/or AD pathologies, focusing on plaque deposition, tau hyperphosphorylation and neuroinflammation.

### FNDC5/Irisin

FNDC5 is a glycosylated type I membrane protein, present in skeletal muscle and adipose tissue. During exercise, FNDC5 undergoes proteolytic cleavage and is released into circulation (47). This cleaved form of FNDC5, named “irisin,” can be detected in both plasma and cerebrospinal fluid (CSF) (48), and its levels change depending on age, sex and underlying pathological conditions impacting skeletal muscle metabolism (48, 49). Furthermore, levels of circulating irisin increase in human and mouse serum post-exercise (38, 48, 50) (Figure 2). Interestingly, circulating levels of FNDC5/irisin were reduced in the CSF and serum of a small cohort of AD patients (38). Exogenous delivery of FNDC5/irisin rescued synaptic plasticity and memory defects in a mouse model of Aβ-peptide hippocampal toxicity (38) and appears to reduce accumulation of hyperphosphorylated tau and increase levels of synaptic proteins in the hippocampus of human Tau transgenic mice (51). However, the mechanisms responsible for these effects remain currently unknown.

In addition to the neurotrophic effects of FNDC5/irisin, recent work has also suggested it may act as a novel potential regulator of neuroinflammation (52), another one of the central hallmarks of AD pathology (1). Peripheral delivery of recombinant irisin protein via intraperitoneal injection was sufficient to reduce levels of pro-neuroinflammatory markers GFAP, interleukin-1β (IL-1β) and interleukin-6 (IL-6) in the CNS of a streptozotocin-induced diabetic mouse model (52). Although whether similar anti-inflammatory effects for FNDC5/irisin occur in models of AD is currently unknown, the Brain Seq-Atlas suggests that the highest levels of expression ofIrisinand its putative receptors are found in astrocytes. This raises the possibility of FNDC5/irisin as a novel regulator of astrogliosis and pro-inflammatory signaling in the CNS, although this hypothesis remains yet to be tested.

It is currently unknown whether the peripheral (circulating) pool of FNDC5/irisin, which is highly responsive to exercise, contributes to the increased levels of FNDC5/irisin in the CNS after exercise. However, adenoviral vector delivery of FNDC5 to the mouse liver elevated circulating irisin levels, which in turn induced expression of BDNF in the hippocampus (50), suggesting that manipulation of peripheral FNDC5/irisin levels is sufficient to activate neurotrophic signaling in the CNS. This highlights the potential of FNDC5/irisin as a novel, non-invasive therapeutic for AD, which is currently an area of hot investigation.

### Cathepsin B

Cathepsin B (CTSB), a lysosomal cysteine protease, is another muscle-originating circulating factor known to be upregulated in skeletal muscle, plasma, and the hippocampus after running in rodents, rhesus monkeys and humans (39) (Figure 2). Like FNDC5/irisin, administration of recombinant cathepsin B to adult hippocampal progenitor cells increases levels of BDNF and expression of doublecortin, a marker for neurogenesis (39). Long-term exercise training modulates peripheral levels CTSB and improves memory in middle-aged men, implicating CTSB as a direct mediator of the neurotrophic effects of exercise (53). In agreement with this, CTSB knock-out mice fail to enhance adult hippocampal neurogenesis and spatial memory function after running (39), suggesting that CTSB is required for the full manifestation of the neurocognitive benefits of exercise. Interestingly, CSTB can also directly degrade Aβ assemblies (54) and lentiviral overexpression of CTSB reduces existing plaque deposits in hAPP mice (55). Cathepsin B also promotes the processing and secretion of mature-IL-1β through activation of microglia (56, 57) in a model of chronic pain. Similar anti-neuroinflammatory effects were detected in a systemic LPS exposure model leading to AD-like phenotypes in the CNS (58), although additional studies clarifying the role of CTSB in neuroinflammation in established AD models are needed.

Like FNDC5/irisin, the precise relationship between peripheral/circulating vs. central/hippocampal levels of CTSB remains currently poorly understood. However, it is interesting that whole-brain levels of CTSB are elevated in CTSB-KO mice after peripheral intravenous injection of recombinant CTSB (39), suggesting that at least some of the peripheral pool of CTSB is permeable through the blood-brain barrier. Cathepsin B thus represents a potentially ideal exercise pharmaco-mimetic target: it is responsive to exercise, can activate hippocampal BDNF signaling and may possesses direct antiamyloidogenic and anti-neuroinflammation properties.

However, it is important to note that the role of CTSB in CNS function is controversial: as a lysosomal protease, increases in CTSB levels have also been associated with neuronal cell death in response to transient ischemia and traumatic brain injury (59). Furthermore, it appears that the specificity of CTSB to cleave APP is markedly reduced in the presence of the APP Swedish mutation, one of the causative genes for familial AD (60). Further studies probing the specific cleavage sites and activity, as well as any potential competition/cooperation between CTSB and other β-secretase-like proteases should help illuminate these apparent contradictory results.

### Interleukin-6

Exercise increases local production and secretion of interleukin-6 (IL-6) in skeletal muscle (61, 62), which is followed by a rapid decline after the end of the exercise period in healthy individuals (61, 63). Currently recognized as an exercise-inducible myokine, the biological output of IL-6 signaling is paradoxical. On one hand, chronically elevated levels of IL-6 appear to be a signal for inflammation, are transduced via a cleaved, soluble ligand-bound IL-6 Receptor (a phenomenon known as “trans” IL-6 signaling) and are associated with most of the pathogenic action of IL-6 in the CNS (64). Increased levels of IL-6 in the aged brain can be neurotoxic and affect cognitive and motivational systems (65). Altered levels of classical pro-inflammatory interleukins is a well-described feature of AD (66–68), with elevations of IL-6 levels found in the blood and brain tissues of AD patients (67, 69). Moreover, the severity of dementia in cognitive disorders like Down syndrome and AD is associated with increased IL-6 levels (70). Further, activated microglia and astrocytes surround amyloid plaques in AD, suggesting neurotoxic effects of cytokines (1). Interestingly, IL-6 is present in these senile plaques and could be detectable prior to amyloid plaque formation (1, 71), suggesting inflammatory cytokines may serve as primers for neuropathological changes as seen in development of AD. Indeed, IL-6 can cross the blood-brain barrier, suggesting that chronic levels of peripheral inflammation may modulate central inflammatory processes and contribute to age-associated neurocognitive decline (ie. the “inflammaging” hypothesis) (12).

Conversely, transient elevations of IL-6, such as those seen during exercise, appear to play an anti-inflammatory role (61, 72) via “classical” IL-6 signaling, where the IL-6 ligand binds to a membrane-bound form of IL-6 receptor. Indeed, some studies have shown that the acute increases in IL-6 levels caused by exercise inhibit the activation of pro-inflammatory factor TNFα (73) and promote anti-inflammatory signaling (Figure 2) (74). Exercise also plays an important role modulating brain cytokine levels, altering the immune profile and improving cognitive performance and decreased amyloid deposition in the brain of Tg2576 Alzheimer's disease transgenic mice (75). Interestingly a recent a study showed that 16 weeks of exercise paradigm in AD patients increases plasma IL-6 in the exercise group, compared to control group (76), suggesting similar exercise-associated muscle IL-6 responses in AD and healthy control groups. Although it is currently unclear whether IL-6 is necessary for the CNS-targeting benefits of exercise, one interesting hypothesis is that transient IL-6 signaling originating from skeletal muscle may favor “classical” over “trans”-IL6 signaling, or perhaps be enriched for different populations of the ten currently identified members of the IL-6 family. Given the underlying elevations in IL-6 levels in circulation in patients with AD, understanding the contributions of acute vs. chronic IL-6 signaling, as well as identifying the downstream effects of exercise-associated vs. aging-associated responses in IL-6 secretion from skeletal muscle will be fundamental in the development of exercise-mimicking therapeutics.

## Exercise-Mimicking Therapeutic Interventions: the AMPK-PGC-1α-PPARβ/Δ Pathway

Exercise is an effective intervention against a wide variety of metabolic problems, age-related loss of function, and physiological complications. However, the benefits of behavioral exercise interventions remain inaccessible to many of the most at-risk populations, either due to economic status, immobility, or advancing disease. Although it is unlikely that a single medication or therapeutic target will achieve the plethora of benefits of exercise on brain and body physiology (77), identification of agents that mimic or potentiate the neurotrophic effects of endurance exercise are a highly attractive target for AD therapeutic development (Figures 1, 2). Multiple studies have focused on the metabolic networks and transcriptional activators involved in the beneficial roles of exercise in skeletal muscle although to date very little is known about their effects on myokine synthesis and secretion.

5' AMP-activated protein kinase (AMPK) is a master regulator of cellular metabolism, facilitating the energetic switch toward cellular catabolism in skeletal muscle during exercise. AMPK activation has both rapid effects induced via direct phosphorylation of downstream metabolic enzymes (metabolic reprogramming), and slower, more sustained effects achieved by transcriptional reprogramming through phosphorylation of PPARγ coactivator 1-alpha (PGC1α), a master regulator of mitochondrial biogenesis (77). Peroxisome proliferator-activated receptor (PPAR) PPARβ/δ is a PGC1α co-factor with known effects on mitochondrial biogenesis, lipid metabolism and oxidative processes, and is also required for the transcriptional adaptations to exercise in skeletal muscle. The skeletal muscle AMPK-PGC-1α pathway is one of the most examined signaling network in the field of exercise adaptations (77), yielding multiple pharmaceutical compounds and genetic interventions that mimic some of the local and CNS benefits of exercise.

For example, systemic administration of a well-known AMPK agonist, 5-Aminoimidazole-4-carboxamide 1-β-D-ribofuranoside (AICAR) to male young C57/BL6 mice activated BDNF and neurogenesis in the dentate gyrus of the hippocampus to levels equivalent to those seen in untreated runners (78). Even in 2-year-old mice, longer AICAR treatment improves memory and motor coordination (79). Interestingly, these effects are likely to be due to peripheral activation of AICAR-sensitive pathways, given the low permeability of this compound through the blood-brain barrier. In agreement with this, the AICAR-associated improvements in spatial memory are lost in muscle-specific AMPKα2 mutant mice (79), suggesting that activation of AMPK signaling may be an essential requirement for the CNS-targeting effects of exercise (Figure 2).

Metformin, another compound believed to activate AMPK signaling and with well-documented metabolic corrective effects, was recently found to act as a neuroprotective agent in a mouse model of AD (80), although these effects were only seen in females. Metformin can also act as a bonafide neuroprotectant, activating neurogenesis, increasing focal angiogenesis, and attenuating ischemia-induced brain injury in mice (81). Supported by lifespan-extension studies in rodents, metformin is currently being tested in human pre-clinical trials as a novel gero-protectant (Metformin in Longevity Study, MILES). Characterization of the biological fingerprint of aging in skeletal muscle before and after metformin treatment may yield important information of metformin-dependent transcriptional reprogramming associated with protection against age-associated disease, including any potential changes in exercise-induced myokines. Furthermore, long-term follow-up of study participants in the metformin arm of the trial may help to determine any potential protection against the development of AD.

PPARδ acts synergistically with PGC1α during exercise-associated muscle remodeling. GW501516 is a well-characterized PPARβ/δ agonist, promoting endurance-associated benefits on skeletal muscle (Figure 2) (77). In addition to multiple metabolism-associated benefits on skeletal muscle and liver of both genetic and induced mouse models of obesity, some evidence also suggests that GW501516 may have also some anti-inflammatory effects (82). Like AICAR, GW501516 has low blood-brain barrier permeability, implicating effects on the periphery as main drivers of the observed metabolic improvements. In agreement with this, important CNS benefits were seen after systemic GW501516 treatment in a mouse model of Huntington's Disease (83), directly implicating peripheral PPARβ/δ as powerful novel neuroprotective pathway from the periphery into the CNS. Interestingly, direct infusion of GW501516 into the CNS may also have neuroprotective effects, activating neurogenesis and enhancing spatial memory in young female mice (84) and reducing both ischemia- and MPTP-induced neuronal toxicity (85). To our knowledge, no studies have examined changes in skeletal muscle-originating exercise induced myokines after GW501516 peripheral treatments but may represent a currently unexplored mechanism for the neuroprotective effects of GW501516 in models of neurodegenerative disease.

It is important to note that manipulation of individual members of the AMPK-PGC-1α pathway sometimes has also failed to yield effects on the CNS. For example, muscle-specific overexpression of PGC1α, a major regulator of the constitutively developed endurance muscle phenotype in rodents, was insufficient in preventing age-associated declines in hippocampal neurogenesis (86). Furthermore, the benefits of AICAR treatment on neurocognitive function in the CNS are transient, disappearing after 2 weeks of treatment. This is likely due to elevations in circulating and local pro-inflammatory cytokines with prolonged AICAR treatment (78). Because of this, understanding the long-term effects of these medications on neurocognitive function in humans—and separating these effects from existing co-morbidities or co-medications—will be essential, particularly for long-standing FDA approved compounds such as metformin.

Indeed, the question of exactly what cellular and biochemical remodeling pathways are activated via these pharmacological and/or genetic interventions remains: for example, although both exercise and GW501516 increase mitochondria fatty acid oxidation and fat metabolism in skeletal muscle, their downstream effects diverge. While exercise acts by promoting catabolism, glycolysis and gluconeogenesis in skeletal muscle, GW501516 treatment regulates branched chain amino acid and ketone body pathways instead (87). These studies suggest that exercise-mimetics that produce isolated changes in muscle consistent with those of exercise, independent of changes in exercise induced myokines profiles, may lead to unwanted counter-productive off target-effects (e.g., inflammation), failing to achieve sustainable neurotrophic effects on the CNS. On-going multi-site studies mapping the dynamic responses to exercise in metabolic tissues, including the brain, in response to different exercise modalities (27), will be essential to identify central pathways for targeted therapeutic developments of exercise-associated rejuvenation of the CNS.

It should be noted that dysregulation of AMPK signaling is prevalent in AD, suggesting that further manipulation of this pathway may, at best, fail to achieve the observed benefits when used in combination of existing AD-associated pathologies and at worst, exacerbate AD-associated symptoms. Further clarification of the specific nodes of AMPK-signaling altered in AD will facilitate therapeutic applications of AICAR and related compounds in AD. Finally, given the sex-specific metabolic dysfunction observed in some AD studies (88) and the sex-differences reported in pre-clinical AMPK activator studies (78, 80), one additional potential area of investigation lies in the study of sex-differences in metabolic-targeting as an AD therapeutic intervention.

## APOE Status and Exercise

Apolipoprotein E (*APOE*) status may modify the threshold for associations between physical exercise and dementia (89). Indeed, multiple lines of evidence suggest stronger protective effects of exercise in carriers of the *APOE* ε4 allele (19, 90–92), one of the highest genetic risk factors for AD, found in almost half of the late-onset AD cases (93). For example, a population based study of more than 1,600 older adults assessing the interaction between *APOE* status and exercise on dementia risk over a 5-year period suggests that physical exercise significantly moderated the relationship between genotype and dementia (94). In a *post-hoc* analysis of 200 patients with mild AD, patients who carried the *APOE4* allele benefited more from the exercise intervention than non-carriers on cognitive, neuropsychiatric, and physical measures, suggesting that behavioral exercise interventions may also provide benefits after the onset of AD (95). Although these results have sometimes not been replicated (96–98) or flat out contradicted (99), we believe this reflects the complex relationship between exercise modalities, AD disease state and *APOE* status. Analysis of myokine profiles (before and after exercise) on APOE populations may yield novel information about skeletal muscle responses in the context of this high-risk gene.

## Current Challenges

The variability of exercise paradigms used in human and animal model interventional trials (100), including high-intensity interval training (HIIT), resistance exercise, aerobic exercise, and multi-modal training, all associated with different intensities and durations, further complicates the identification of an “exercise prescription” for targeted therapeutic effects. Unification of duration and intensity of exercise-interventions in future clinical studies will be essential to allow comparisons across genotype, age, and disease progression. Indeed, human studies have demonstrated clear individual variations in post-exercise training metabolic adaptations (101), and individuals that already had cognitive impairments require higher levels of physical activity to achieve similar neurocognitive benefits (19). Inclusion of other factors including baseline fitness, dietary factors, training history, and between-session recovery during trials may also help explain these seemingly contradictory results (27). Finally, the historical underrepresentation of both women and non-Caucasian populations on exercise trials has prevented our understanding of the role of hormones and genetic diversity on exercise responses. On-going efforts to characterize tissue responses to these different exercise modalities in human populations and murine models (27) will yield key information for future behavioral exercise interventions with CNS benefits.

The benefits of physical activity and/or exercise are numerous and well-documented, although the precise mechanisms responsible for the observed improvements in cardiovascular, neurocognitive and well-being measurements remain poorly understood (Figure 1) (27). However, even if exercise interventions, be it behavioral or pharmacological, do not alter the known pathological hallmarks of AD, their use as adjuvants to correct downstream biological processes in combination with other therapeutic interventions targeting AD pathological findings is highly attractive from a clinical and financial standpoint. If exercise interventions could delay the progression of AD and impact nursing home placement and hospice care, for example, would lead to major benefits for patients, caretakers and medical personnel in the fight against AD.

## Conclusion

The number of identified circulating factors produced and released from exercising skeletal muscle continues to grow. Indeed, the skeletal muscle secretome contains both free and membrane-bound bioactive factors, including proteins, metabolites, and micro-RNAs. Furthermore, the effects of exercise on the muscle secretome profile are only barely beginning to be understood. The construction of an atlas of exercise-responsive myokine or myokine-associated nodes through the on-going Molecular Transducers of Physical Activity Consortium (MoTRPAC) studies (27), as well as specific modifications to these networks with aging or disease (including AD), will inform potential therapeutic interventions that reproduce the neuroprotective effects of exercise. Locally, exercise pharmaco-mimetics should reproduce the local effects on skeletal muscle, including re-programming the established muscle fiber specification toward an overt endurance phenotype, and activation of mitochondrial proteostasis and functional pathways. They should reproduce similar myokine profiles in circulation and ideally be orally active. Improving CNS resilience during aging, either through behavioral, pharmacological, or combinatorial approaches, should thus be a high-priority target for our field. Importantly, this will require integrated efforts of multiple scientific communities, including neuroscientists, clinicians, and exercise physiologists.

## Author Contributions

RG, RK, and CC worked together in the development of this manuscript and wrote the initial drafts of the manuscript. RG and RK performed literature searches and created the associated figures, with direct input and supervision from CC. CC edited and compiled the final version.

## Conflict of Interest

The authors declare that the research was conducted in the absence of any commercial or financial relationships that could be construed as a potential conflict of interest.
